# Determination of *para*-Phenylenediamine (PPD) in Henna in the United Arab Emirates

**DOI:** 10.3390/ijerph7041681

**Published:** 2010-04-13

**Authors:** Ayesha Al-Suwaidi, Hafiz Ahmed

**Affiliations:** 1 Environment Management Sector, Environment Agency-Abu Dhabi, UAE; E-Mail: Ayesha.AlSuwaidi@ead.ae; 2 Department of Environmental Health, College of Health Sciences, University of Sharjah, P.O. Box 27272, UAE

**Keywords:** henna, *para*-phenylenediamine, contact allergy, G6PD deficiency, renal failure, UAE

## Abstract

Henna is very popular in the United Arab Emirates (UAE); it is part of the culture and traditions. Allergy to natural henna is not usual; however the addition of *para*-phenylenediamine (PPD) to the natural henna increases the risk of allergic contact dermatitis. The objectives of the study were to identify the presence and concentration of PPD in henna available in UAE. Fifteen henna salons were selected randomly from three cities in UAE. Twenty five henna samples were acquired from these selected salons. The presence of PPD in henna samples was determined qualitatively and quantitatively using High Performance Liquid Chromatography (HPLC). The study showed that PPD was present in all of the black henna samples at concentrations ranging between 0.4% and 29.5% and higher than that recommended for hair dyes in most of the black henna samples. The presence of PPD in the black henna increases the risk of allergic contact dermatitis among users of black henna and a number of cases have already been reported in UAE.

## 1. Introduction

Henna or hina (*Lawsonia inermis*, family Lythraceae) is a flowering plant or shrub native to tropical and subtropical regions of Africa, and Southern Asia. Henna is commercially cultivated in Morocco, Sudan, India, Pakistan, Yemen, and other countries. Henna contains a burgundy dye molecule, lawsone (2-hydroxy-1,4-naphthoquinone). This dye molecule has the ability to bond with proteins, and consequently has been widely used in body art to dye skin, hair and fingernails, and to dye silk, leather, and wool. Henna body art is made by applying henna paste to the skin. Henna paste is prepared by drying the henna leaves and grinding them to powder, and then this powder is mixed with oil or water to form the paste. When this henna paste is applied to the skin the dye (lawsone) migrates from the paste to the outermost layer of the skin; more lawsone will migrate if the paste is left on the skin for a longer time, thus creating a red-brown stain [1].

Henna has been used to adorn women’s bodies during marriage ceremonies and other social celebrations since the Bronze Age. In the Arab world and Indian subcontinent henna is used for skin decoration and hair dying during social celebrations, and during marriage ceremonies people celebrate by adorning the bride, and sometimes the groom, with henna [1].

Despite the wide spread use of natural henna, specially, in countries where henna art is traditionally practiced, reports of allergic contact dermatitis to natural henna are very rare in the literature. It can therefore be assumed that natural henna is a very weak skin allergen [2].

*para*-Phenylenediamine (PPD) is an aromatic amine compound; its chemical formula is C_6_H_8_N_2_ and its molecular weight is 108.15 g/mol. It is white to light purple powder that darkens on exposure to air (it oxidizes, turning first red, then brown then finally black); it is slightly soluble in water [3–5]. It is primarily used as an ingredient of oxidative hair coloring products at a maximal concentration of 4.0%, however, after mixing in a 1:1 ratio with hydrogen peroxide prior to use this concentration will be 2% at the time of application to the hair. In addition to hair dyes, PPD may also be found in fur or textile dyes [5]. *para*-Phenylenediamine is also used as a photographic developing agent as well as an antioxidant in rubber compounds. Individuals may be occupationally exposed to PPD during its manufacture or use, and the exposure may occur through inhalation, skin and/or eye contact, and ingestion [3–5].

Short-term exposure to high levels of PPD (acute effects) may cause severe dermatitis, eye irritation and tearing, asthma, gastritis, renal failure, vertigo, tremors, convulsions, and coma in humans. Eczematous contact dermatitis may result from long-term exposure (chronic effect) in humans [3–8]. According to Scientific Committee on Consumer Products (SCCP), *para*-phenylenediamine is a very strong potential skin sensitizer and it is included as such in the European Standard Series for diagnostic patch testing for eczema patients [5]. *para*-Phenylenediamine (PPD) is an allergen; even if someone does not have a reaction the first time they are exposed to it, they can become “sensitized” to PPD over time and can have adverse reaction upon re-exposure [9].

In addition, PPD provokes cross-allergy, making people allergic to other substances which contain *para*-substituted amino compounds [6]. No information is available on the reproductive, developmental, or carcinogenic effects of PPD in humans [3]. However, SCCP reported that PPD together with hydrogen peroxide may be carcinogenic according to experimental studies with rats [5].

Recently *para*-phenylenediamine has been mixed with natural henna to give an ebony color (black henna) instead of the orange/reddish color given by natural henna. The other reason for adding PPD to the natural henna is to speed up (shorten the time) of the tattooing process, while natural henna staining takes 4 to 12 hours, addition of PPD can reduce this time to an hour or two and also there will be a longer lasting effect as well. Thus, a new pattern of exposure to PPD has been recognized through henna art which increases the risk of developing adverse health effects related to PPD [7]. Acute allergic contact dermatitis, eczema, chemical burn, acute renal failure, acute and severe angioneurotic edema, abdominal pain and vomiting as adverse health effects associated with the use of henna containing PPD (black henna) are well documented in the literature [10–16]. In addition, cases of persons being sensitized from use of black henna (containing PPD) followed by cross reaction to oxidative hair dyes and to clothing dyes have also been described in the literature [17,18].

In the UAE, the use of henna is part of the tradition and culture. Women of all ages use henna for skin decoration, and it is considered an essential part of the wedding ceremonies and other social celebrations. Thus the objectives of the study were to identify the presence and concentration of PPD in black and red henna available in UAE.

## 2. Materials and Methods

### 2.1. Samples

Proportional random sampling was used to select 15 henna salons from three cities in the UAE. Twenty five henna samples were bought from these selected salons, 11 of them were black henna and the remaining 14 were red.

### 2.2. Analysis of Samples for PPD

PPD standard (0.11 mg per mL) was prepared by weighing pure PPD substance (Sigma Life Science, 0.011 gram) and dissolving it in 50 % aqueous methanol solution (100 mL). High-performance Liquid Chromatography (HPLC) was performed using an Agilent 1100 HPLC Series equipped with a Refractive Index Detector (RID) with a wavelength of 290 nm and a spectrum range of 190 to 400 nm. The HPLC conditions were: mobile phase, 0.05 M acetic acid-methanol (95/5) and adjusted to a pH of 5.9 with ammonia; the temperature was 30°C; flow rate was 1.5 mL/min; wave- length 290 nm; pressure 174 bar; and column, LiChrospher RP18 5μm, 250 mm × 4.6 mm.

One gram of each of the collected samples was weighed into a 50 mL volumetric flask and diluted with 50% aqueous methanol solution (50 mL). This solution was then filtered after 15 minutes. Finally one mL of this solution was diluted to 5 mL with 50% aqueous methanol solution and analyzed for PPD.

To confirm the identity of PPD in our samples, one mL of the standard was diluted to 5 mL with 50% aqueous methanol solution and analyzed before analyzing any sample to determine its spectrum and its retention time.

## 3. Results and Discussion

### 3.1. PPD Concentration in Samples

**Figure 1.**
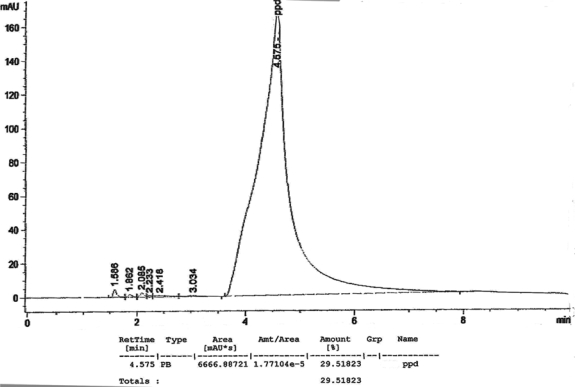
Chromatogram of PPD in black henna sample 1.

**Figure 2.**
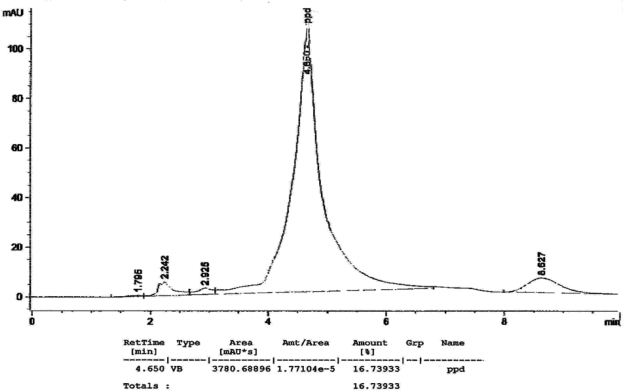
Chromatogram of PPD in black henna sample 2.

**Figure 3.**
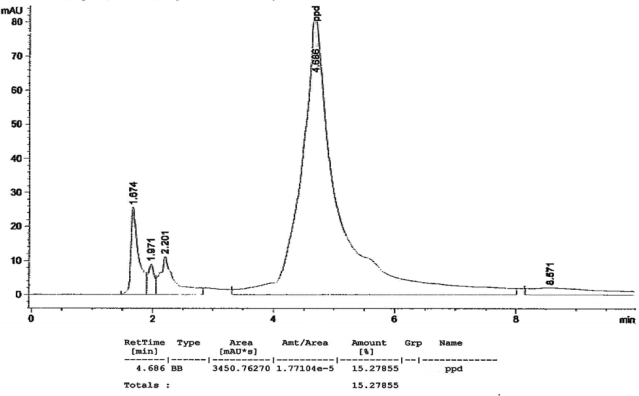
Chromatogram of PPD in black henna sample 3.

**Figure 4.**
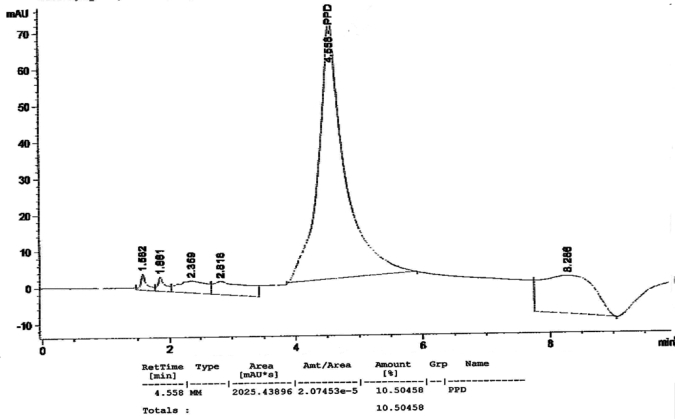
Chromatogram of PPD in black henna sample 4.

**Figure 5.**
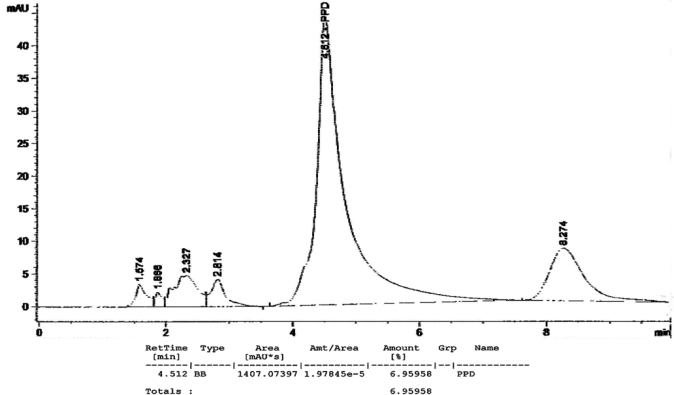
Chromatogram of PPD in black henna sample 5.

**Figure 6.**
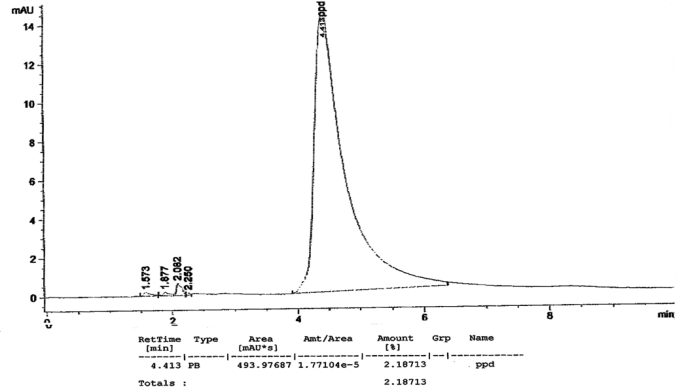
Chromatogram of PPD in black henna sample 6.

**Figure 7.**
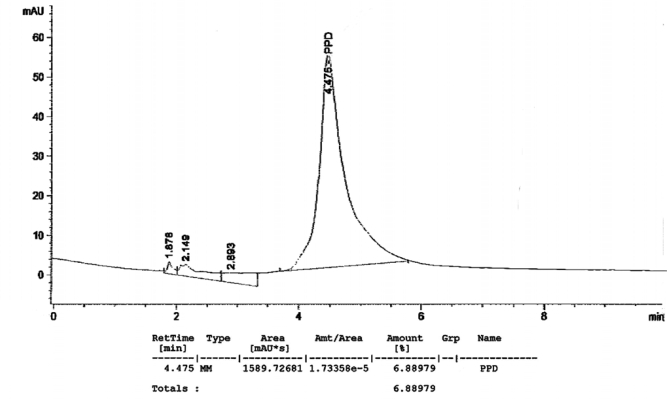
Chromatogram of PPD in black henna sample 7.

**Figure 8.**
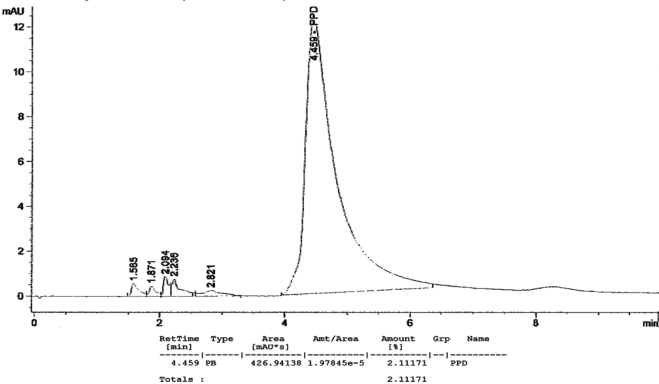
Chromatogram of PPD in black henna sample 8.

**Figure 9.**
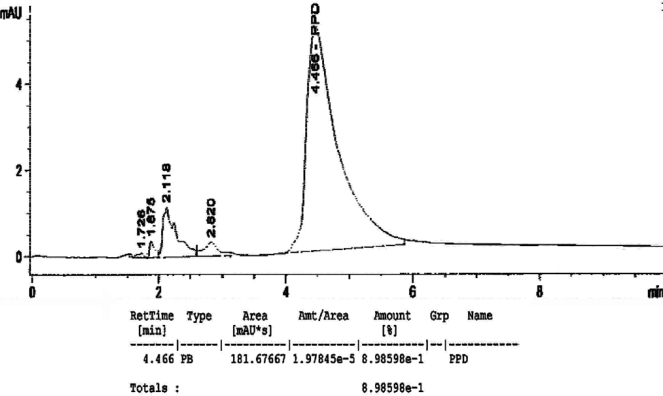
Chromatogram of PPD in black henna sample 9.

**Figure 10.**
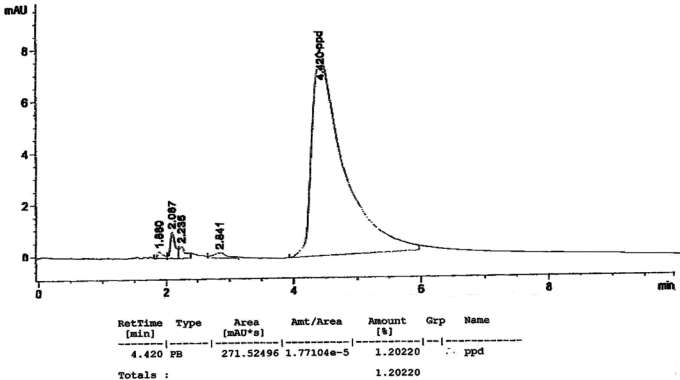
Chromatogram of PPD in black henna sample 10.

Figures 1 to 10 show the chromatograms of the PPD in black henna samples. The retention time for the PPD standard was found to be 4.565 minutes and under the same analysis conditions the retention times for the samples were found to range between 4.413 and 4.686 minutes. In addition, the spectrum of each of our samples was found to be a good match with that of the standard, confirming that the peak and the retention time of the samples was the same as that of the PPD standard. The concentration of PPD in each sample was read directly from the HPLC.

As shown in Table 1 and Figures 1–10. The PPD was found in all the black henna samples (11 samples) with concentration ranging between 0.38 % and 29.5%. The concentration of PPD was more than 10% in four black henna samples, more than 6% in six samples, more than 2% in eight samples, and less than 1% in two black henna samples. For red henna samples, PPD was not detected in nine samples and for the remaining five red henna samples the concentration of PPD was very low, ranging between 0.005% and 0.23%.

The use of HPLC permits PPD characterization by its spectrum and retention time, avoiding any risk of false identification of the substance. Thus, HPLC allowed the determination of PPD in henna samples both qualitatively and quantitatively. Moreover, the highest concentration of PPD reported by this study (29.5%) was significantly higher than that reported by Brancaccio and his colleagues in their study (15.7%) [12], and also higher than that reported by Kang and Lee (2.35%) [19].

When comparing the concentration of PPD in black henna to that in red henna we found that the concentration of PPD in black Henna was as expected higher because the black color indicates a high concentration of PPD.

The concentration of PPD in eight black henna samples was higher than the maximum concentration of PPD found in hair dyes when applied to hair as specified by SCCP which is 2.0% [5]. The concentration of PPD reported by this study for six black henna samples was higher than the permitted concentration of PPD in hair dye products established by the European Union, which is 6.0% [12], and this finding is consistent with other researchers who found the level of PPD in henna tattoos was much higher than that found in hair color [12–15]. However, the European Union has banned the use of PPD directly on the skin, eyelashes or eyebrows, and Food and Drug Administration (FDA) has prohibited the use of PPD directly on the skin [12].

### 3.2. Cases

Many cases of contact allergy have been reported in UAE after the use of Black Henna. Picture 1 shows one of these cases where the patient has developed hand sensitization few days after the use of black henna.

Picture 2 shows a positive result for a patch test done on the patient with a PPD concentration of 0.1 [20]. In addition, a case was reported in one of the hospitals in UAE. The patient suffered from vomiting and abdominal pain after the laboratory test she was diagnosed with poisoning due to henna. The patient also had glucose-6-phosphate dehydrogenase (G6PD) enzyme deficiency, a genetic disease which is more common in the Gulf region [20]. Usually, G6PD deficiency leads to an abnormal rupture (breakage) of the red blood cells called haemolytic anemia. There is evidence that many substances are potentially harmful to people with G6PD deficiency and they can cause acute haemolysis in people with G6PD deficiency. These substances include: anti-malarial medications (such as chloroquine, primaquine), sulfonamides (such as sulfamethoxazole, sulfanilamide). PPD has a similar structure to these substances and thus it might cause the same effect in people with G6PD deficiency.

In addition, it is reported in the literature that henna has been known to cause haemolytic crisis in G6PD-deficient infants and others [21,22]. In the UAE, Raupp and his colleagues have reported the occurrence of haemolytic crisis in four G6PD-deficient children following topical application of henna; of these a female neonate recovered after exchange transfusion, and a male infant died despite receiving a transfusion [23].

## 4. Conclusions and Recommendations

The study shows that the samples of black henna bought from the henna salons included in this study contained high concentration of *para*-phenylenediamine, and this increases the risk of sensitization among those using black henna. People who have developed contact dermatitis from the use of black henna and those have not are both susceptible to contact allergy from henna mixed with PPD. In addition, people developed sensitization from use of black henna are susceptible to cross reaction to oxidative hair dyes and to clothing dyes. The cases reported in the UAE hospitals confirm the conclusion that henna mixed with PPD (black henna) could have adverse health effects on the human health. Based on the findings of this study the investigators recommended that addition of PPD to natural henna should be prohibited in UAE. In particular people who have glucose-6-phosphate dehydrogenase (G6PD) deficiency should be informed about the potentially serious adverse health effects of topical application of henna. In addition, an awareness program should be established to inform the public about the risk of using henna mixed with PPD (black henna).

## Figures and Tables

**Picture 1.**
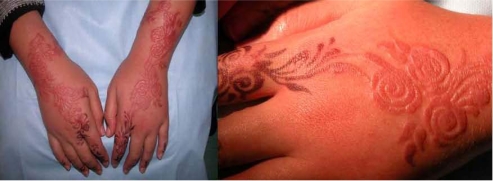
Hand dermatitis, reaction to black henna.

**Picture 2.**
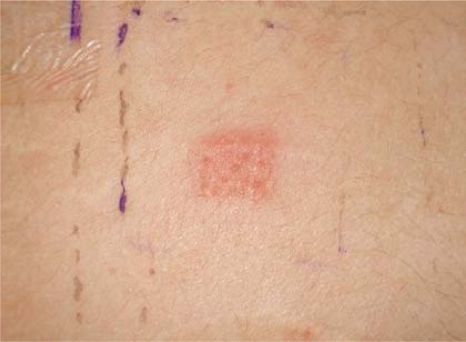
Positive reaction to patch test done using 0.1% PPD.

**Table 1.** The Concentration of *para*-Phenylenediamine in Henna Samples.

**City**	**Salon No.**	**Black Henna**		**Red Henna**	
		**Sample No.**	**PPD (%)**	**Sample No.**	**PPD (%)**
A	1	1	29.518	-	-
A	2	1	16.739	1	ND*
A	2	2	15.279	-	-
A	2	3	10.505	-	-
A	2	4	6.960	-	-
A	3	1	2.187	1	ND*
A	4	1	6.890	1	ND*
A	5	-	-	1	0.012
A	6	-	-	1	ND*
B	1	1	2.112	1	ND*
B	2	-	-	1	0.005
B	3	-	-	1	0.007
B	4	1	0.899	1	ND*
B	5	-	-	1	ND*
C	1	1	1.202	1	0.038
C	2	-	-	1	0.230
C	3	1	0.383	1	ND*
C	4	-	-	1	ND*

ND* = Not Detected.
